# Ultrasensitive Detection of Rare Mutations via Amplifying–Cleaving–Enriching in Acute Myeloid Leukemia

**DOI:** 10.3390/biomedicines13123026

**Published:** 2025-12-10

**Authors:** Xiaomei Zhuang, Lingling Ma, Liuting Yu, Yuming Zhao, Dengyang Zhang, Chunmou Li, Chaoxing Liu, Yan Xiao, Zhiguang Chang, Shuping Li, Chun Chen, Yun Chen, Guoying Zhou, Zhizhuang Joe Zhao, Yao Guo

**Affiliations:** 1 Pediatric Hematology Laboratory, Division of Hematology/Oncology, Department of Pediatrics, The Seventh Affiliated Hospital of Sun Yat-sen University, Shenzhen 518107, China; zhuangxm7@mail2.sysu.edu.cn (X.Z.); malling5@mail2.sysu.edu.cn (L.M.); yult3@mail.sysu.edu.cn (L.Y.); zhaoym29@mail.sysu.edu.cn (Y.Z.); zhangdy39@mail.sysu.edu.cn (D.Z.); lichunmou1@sysush.com (C.L.); liuchx69@mail.sysu.edu.cn (C.L.); xiaoy357@mail2.sysu.edu.cn (Y.X.); changzhg@mail.sysu.edu.cn (Z.C.); chenchun@mail.sysu.edu.cn (C.C.); cheny653@mail.sysu.edu.cn (Y.C.); 2 Digestive Diseases Center, The Seventh Affiliated Hospital of Sun Yat-sen University, Shenzhen 518107, China; 3 Guangdong Provincial Key Laboratory of Digestive Cancer Research, The Seventh Affiliated Hospital of Sun Yat-sen University, Shenzhen 518107, China; zhougy9@mail.sysu.edu.cn; 4 Department of Pathology, University of Oklahoma Health Sciences Center, 940 Stanton L. Young Blvd., BMSB 451, Oklahoma City, OK 73104, USA; scorpiolishuping@163.com; 5 Scientific Research Center, The Seventh Affiliated Hospital of Sun Yat-sen University, Shenzhen 518107, China

**Keywords:** AML, detection of mutations, FLT3-TKD, IDH1

## Abstract

**Background:** Detecting low-frequency mutations is crucial for predicting prognosis and monitoring minimal residual disease (MRD) in acute myeloid leukemia (AML). However, the presence of abundant wild-type sequences hinders the detection of rare mutant alleles. We present a highly sensitive method called ACE (Amplifying–Cleaving–Enriching) to selectively enrich mutant sequences. **Methods:** ACE includes three steps: (1) initial PCR amplification using biotin-labeled primers, (2) cleavage of wild-type sequences with a specific restriction enzyme, and (3) enrichment of undigested mutant alleles via streptavidin-labeled magnetic beads. **Results:** Using two rounds of ACE, we achieved over 80,000-fold enrichment of mutant sequences carrying FLT3-TKD, enabling the detection of mutant alleles at levels as low as 0.0001% in AML patient blood samples. Additionally, the ACE method can be adapted to nearly any driver mutation by introducing wild-type-specific restriction sites through PCR with mismatched primers, which has been validated in the IDH1 mutation. Furthermore, the ACE method can be flexibly integrated into conventional detection techniques including Sanger sequencing, quantitative real-time PCR, allele-specific PCR, and even with advanced techniques like droplet digital PCR. **Conclusions**: ACE significantly enhances the sensitivity of existing techniques for rare mutation detection and holds potential for broad clinical applications.

## 1. Introduction

Acute myeloid leukemia (AML) is a highly heterogeneous hematopoietic malignancy characterized by the clonal expansion and accumulation of myeloblasts in the bone marrow and peripheral blood [1]. This leads to impaired normal hematopoiesis and rapid disease progression. AML affects individuals across all age groups but is more prevalent in older adults, often resulting in a poor prognosis [2]. While most patients achieve remission after cytotoxic chemotherapy [3], the disease frequently relapses, particularly in those with high-risk genetic profiles or poor initial response to treatment [4]. Therefore, accurate risk stratification and close monitoring for minimal residual disease (MRD) are critical for improving patient outcomes and guiding therapeutic strategies.

Over 20 recurrently mutated genes have been identified in AML, many of which serve as prognostic markers or therapeutic targets [5]. For example, gain-of-function mutations in FLT3 are associated with poor prognosis and lead to the constitutive activation of downstream signaling pathways that promote cell survival and proliferation. This has led to the development of targeted therapies such as midostaurin and gilteritinib, small-molecule inhibitors approved for the treatment of FLT3-mutant AML [6,7,8]. Mutations in IDH1 and IDH2 are another important class of genetic alterations in AML, resulting in the production of the oncometabolite R-2-hydroxyglutarate (R-2HG), which disrupts normal DNA methylation and contributes to leukemogenesis [9,10]. The FDA-approved inhibitors ivosidenib and enasidenib target these mutant enzymes in patients with relapsed or refractory AML, providing clinical benefits [11]. However, resistance to these therapies frequently develops through diverse mechanisms [11,12,13]. Therefore, a highly sensitive and convenient method for detecting these molecular alterations is urgently needed to evaluate MRD and guide therapeutic decisions in AML.

Clinically available techniques for detecting mutations in AML include Sanger sequencing, restriction enzyme fragmentation, next-generation sequencing (NGS), and real-time polymerase chain reaction (PCR) [14]. While these methods have proven valuable, they each come with limitations. Some, like Sanger sequencing, lack the sensitivity needed to detect minimal residual disease (MRD), while others, such as NGS, are expensive, labor-intensive, and time-consuming. A major challenge across these techniques is the overwhelming presence of wild-type sequences, which hampers the detection of low-frequency mutant alleles. To address these issues, we developed a simple and efficient method called ACE (Amplifying–Cleaving–Enriching). This technique selectively enriches mutant sequences by combining wild-type-specific restriction enzyme digestion with streptavidin–biotin affinity isolation. In this study, we evaluated the enrichment efficiency of ACE for FLT3-D835Y/H mutations, which possess a native restriction enzyme cleavage site, and IDH1-R132H mutations, for which we introduced an additional cleavage site via PCR with mismatched primers.

## 2. Methods

### 2.1. Sample Collection and DNA Extraction

Blood samples were collected from patients at the Seventh Affiliated Hospital of Sun Yat-sen University. Blood samples were obtained from two healthy donors and twenty-five AML patients who provided written informed consent using a protocol approved by the Ethics Committee of The Seventh Affiliated Hospital of Sun Yat-sen University (approval number: KY-2024-200-01), in accordance with the Declaration of Helsinki. All samples are uniformly coded by a third party not involved in the experiment to conceal their clinical information, including patient identity, group assignment, treatment phase, etc. Genomic DNA was extracted using the TaKaRa MiniBEST Universal Genomic DNA Extraction Kit Ver.5.0 (TaKaRa, Shiga, Japan). Approximately 1 μg of total DNA was used for each PCR reaction described below.

### 2.2. Plasmid DNA Standards Preparation

The preparation of DNA standards for FLT3-D835 has been previously described [15]. For IDH1-R132H, a 707 bp DNA fragment covering the R132 region of IDH1 was amplified from genomic DNA using primers D1_5 and D1_3 (see Table S1), and subsequently cloned into the pBluescriptSK vector (Agilent Technologies, Santa Clara, CA, USA). Plasmid DNA was purified from *E. coli* using the TaKaRa MiniBEST Plasmid Purification Kit Ver.4.0 (TaKaRa, Shiga, Japan). The IDH1-R132H mutant plasmid was generated via PCR mutagenesis using the Q5^®^ Site-Directed Mutagenesis Kit (New England Biolabs, Ipswich, MA, USA) with primers D1_Hm5 and D1_Hm3 (Table S1). All plasmid constructs were sequence-verified, mixed at varying ratios, and diluted to a final concentration of 10 μg/mL. For PCR analysis, 1 μL of the DNA mixtures was used. DNA concentrations were measured using a NanoDrop™ One spectrophotometer (Thermo Fisher Scientific, Waltham, MA, USA) at 260 nm.

### 2.3. Initial and Derived Cleaved Amplified Polymorphic Sequences PCR

Isolated plasmids or genomic DNA from blood samples were used as templates for either initial PCR or derived cleaved amplified polymorphic sequences (dCAPS) PCR. For the FLT3 sequence, which contains EcoRV or XhoI restriction enzyme sites in the D835 region, initial PCR was performed using primers F1_5 and F1_3. The PCR reaction was carried out in 20 μL volumes, containing 0.25 μM of each primer, 10 ng of vector DNA or 1 μg of genomic DNA, and 10 μL of PfuUltra II Hotstart PCR Master Mix (Agilent Technologies, Santa Clara, CA, USA). The reaction protocol included 25 cycles of denaturation at 95 °C for 20 s, annealing at 60 °C for 20 s, and extension at 72 °C for 15 s.

For the IDH1 sequence, dCAPS PCR was employed to create a wild-type-specific restriction enzyme cleavage site using a mismatched forward primer (ID_PvuI or ID_BceAI) and reverse primer ID_r. The mismatched forward primers were designed following principles as: (1) The 5′ end was modified by biotin; (2) the mismatch site of forward primer should be designed at least 1–3 bases away from the 3′ end, because PCR amplification initiation highly depends on the perfect pairing between the 3′ end of the primer and the template DNA; and (3) the wild-type-specific restriction enzyme cleavage site should be positioned between the biotin modification and the nucleotide polymorphism site, ensuring that the cleaved fragment containing the wild-type site can be washed away. An online tool named as ‘dCAPS Finder 2.0’ can be exploited to design dCAPS primers (http://helix.wustl.edu/dcaps/dcaps.html, accessed on 2 December 2025). It is necessary to use tools such as NCBI BLAST (BLAST+ version 2.17.0) to compare the entire target genome, ensuring that the restriction enzyme site we plan to introduce is unique within the genome, which avoids off-target cutting and ensures the wild-type specificity at the source. The selection of DNA polymerase is also important; high-fidelity DNA polymerase should not be generated, because the introduced mismatched site by dCAPS forward primer would be cleaved by the unique 3′ → 5′ exonuclease activity of high-fidelity DNA polymerase. The dCAPS PCR was conducted in 25 μL reaction volumes with 0.25 μM of each primer, 10 ng of vector DNA, and 12.5 μL of Taq 2× Master Mix (NEB). The cycling conditions consisted of 25 cycles of 95 °C for 30 s, 56 °C for 30 s, and 68 °C for 20 s.

The initial and dCAPS PCR products were resolved on 3% agarose and visualized with Gel Red (Absin, Shanghai, China), ensuring the specificity and efficiency of amplification. The sequences of all primers used in this study are listed in Supplementary Table S1.

### 2.4. Restriction Enzyme Cleavage

For FLT3 amplicons, digestion was performed in a 10 μL reaction mixture containing 1 μL of PCR product, 20 units of EcoRV (New England Biolabs, Ipswich, MA, USA), 100 units of XhoI (New England Biolabs, Ipswich, MA, USA), and 1 μL of 10× rCutSmart buffer (New England Biolabs, Ipswich, MA, USA). The reaction was incubated at 37 °C for 1 h, followed by 20 min at 65 °C to terminate the digestion. For IDH1 amplicons, the products were digested in a 10 μL reaction mixture containing 1 μL of PCR product, 20 units of HF-PvuI or BceAI (New England Biolabs, Ipswich, MA, USA), and 1 μL of 10× rCutSmart buffer. The reaction was incubated at 37 °C for 15 min, followed by 20 min at 65 °C to terminate the digestion. The digestive products were then resolved on 3% agarose and visualized with Gel Red (Absin, Shanghai, China), ensuring the specificity of restriction enzyme cleavage.

### 2.5. Magnetic Isolation

Dynabeads™ Streptavidin Trial Kit (Thermo Fisher Scientific, Waltham, MA, USA) and M-270 Streptavidin magnetic beads (Thermo Fisher Scientific, Waltham, MA, USA) were employed for the isolation of biotin-labeled DNA fragments, following the manufacturer’s protocol. Briefly, 50 μg of Streptavidin magnetic beads were first washed and then added to a buffer solution containing 10 μL of the digestion products and 10 μL of 2× washing buffer (10 mM Tris-HCl, 1 mM EDTA, 4 M NaCl). The mixture was incubated at room temperature with gentle rotation to facilitate binding between the beads and the biotin-labeled DNA fragments. The magnetic beads were then washed twice to remove unbound material, and the bead-bound DNA fragments were used as templates for the subsequent PCR reactions.

### 2.6. Sanger Sequencing

Amplification of DNA fragments containing the FLT3-D835 region was performed in 40 μL reaction volumes using 20 μL of PfuUltra II Hotstart PCR Master Mix (Agilent Technologies, Santa Clara, CA, USA), 1 μg of genomic DNA, 10 ng of vector DNA, or 5 μL of ACE-enriched product, and 0.5 μM of each primer (F1_5n/F1_3n). The PCR cycling conditions included an initial denaturation at 95 °C for 2 min, followed by 40 cycles of 95 °C for 30 s, 55 °C for 30 s, and 68 °C for 30 s, with a final extension at 72 °C for 3 min.

Similarly, amplification of DNA fragments containing the IDH1-R132 region was performed in 40 μL volumes using Taq 2× Master Mix (New England Biolabs, Ipswich, MA, USA), 0.5 μM of each primer (ID_PvuI/ID_r or ID_BceAI/ID_r), and 10 ng of vector DNA or 5 μL of ACE-enriched products. The thermal cycling conditions consisted of an initial denaturation at 95 °C for 3 min, followed by 40 cycles of 95 °C for 30 s, 56 °C for 30 s, and 68 °C for 20 s, with a final extension at 68 °C for 2 min.

All PCR products were resolved on a 3.0% agarose gel before being sent for Sanger sequencing, which was performed by Genewiz (Suzhou, China). Primer sequences are listed in Supplementary Table S1.

### 2.7. Quantitative Real-Time PCR and Allele-Specific PCR

Quantitative real-time PCR (qPCR) assays were performed using 2× Hieff^®^ qPCR SYBR Green Master Mix (Yeasen, Shanghai, China) to validate the enriched mutant products. Reactions were run on a CFX96™ Real-Time System (Bio-Rad Laboratories, Hercules, CA, USA), with primers at final concentrations of 100 nM. Specific primers F1_5Y/F1_3n, F1_5H/F1_3n, and F1_wt/F1_3n were individually designed to detect FLT3-D835Y, FLT3-D835H, and FLT3-WT, respectively. For improving the reaction specificity and allele discrimination, the mutant site was positioned at the end of the forward primer and a mismatched base was designed at the antepenultimate base of the forward primer. Each 20 μL reaction contained 1 μg of genomic DNA, 10 ng of vector DNA, or 5 μL of ACE-enriched products as templates. The thermal cycling conditions included an activation step at 95 °C for 3 min, followed by 45 cycles of 95 °C for 5 s and 60 °C for 30 s. Primer sequences are listed in Supplementary Table S1.

To quantify the enriched mutant products, a series of standard templates with varying proportions of wild-type and mutant fragments (ranging from 100% to 0%) was prepared. The enriched samples were analyzed using qPCR, and the copy numbers were determined based on standard curves. The enrichment percentage and the fraction of mutant alleles were calculated as the ratio of the mutant fragment copy numbers to the total fragment copy numbers. Allele-specific PCR for detecting FLT3-D835 was previously described [15].

### 2.8. Droplet Digital PCR Assay

Digital PCR (ddPCR) assays were performed using Sniper dPCR mix (Sniper, Suzhou, China) to validate the enriched mutant products. Reactions were run on a Sniper DQ24 series system (Sniper, Suzhou, China), with primers at final concentrations of 500 nM and probes at final concentrations of 250 nM. Specific primers F_TAQ_5 and F_TAQ_3, and probes named Probe_WT (labeled with FAM) and Probe_DY (labeled with VIC) were designed to detect FLT3-D835Y. Each 22 μL reaction contained 0.0001 ng of plasmid DNA or 0.2 μL of ACE-enriched products as templates. The thermal cycling conditions included a stage hold at 60 °C for 5 min and 95 °C for 15 min, followed by 40 cycles of 95 °C for 20 s and 56 °C for 30 s, and a stage hold at 60 °C for 60 s. Primer and probe sequences are listed in Supplementary Table S1.

The mutant fraction was calculated by the proportion of VIC-labeled alleles among the sum of VIC-labeled alleles and FAM-labeled alleles.

### 2.9. Statistical Analyses

The experimental operators and result interpreters are both unaware of the background information of the samples. Set up a negative control for each batch of experiments. Data are presented as the mean ± standard deviation (SD). Differences between two groups were assessed using a 2-tailed Student’s *t*-test. Standard curves were generated using nonlinear regression. Statistical significance was analyzed using GraphPad Prism 6.0 (GraphPad Software Inc., San Diego, CA, USA), and *p*-values less than 0.05 were considered statistically significant.

## 3. Results

### 3.1. Design of Amplifying–Cleaving–Enriching (ACE)

**Figure 1:**
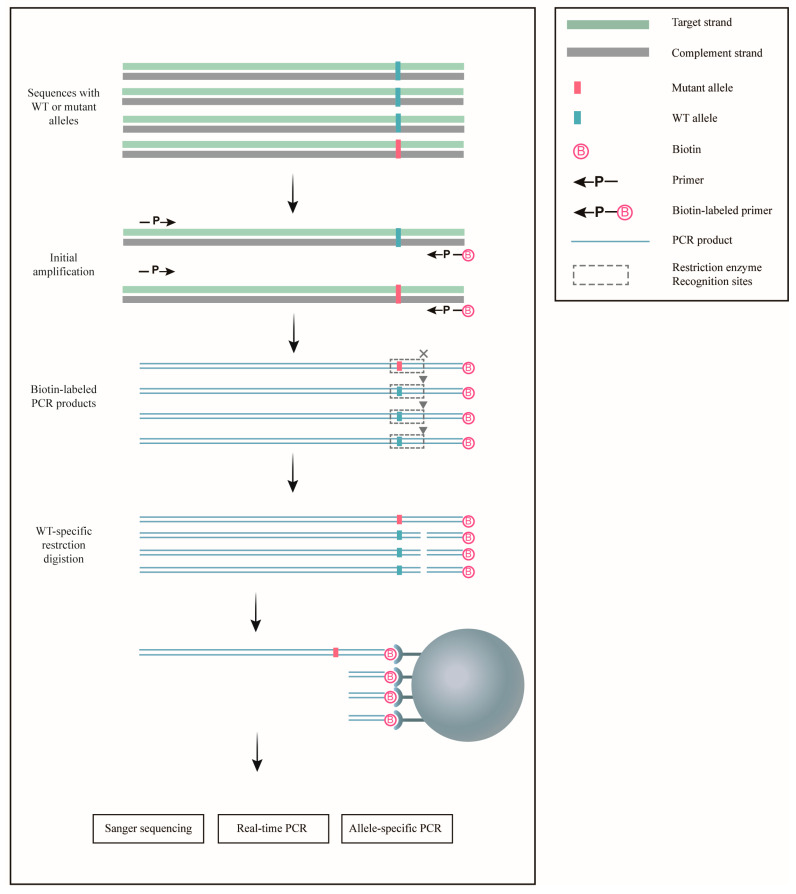
Schematic representation of ACE for the enrichment of rare mutations. Sequences containing either wild-type (WT) or mutant alleles were initially amplified using biotin-labeled primers. The resulting PCR products were then digested with restriction enzymes, which specifically cleaved the wild-type sequences. Streptavidin-coated Dynabeads were employed to isolate the digested products, leaving the undigested mutant allele-containing sequences enriched. These enriched mutant sequences can subsequently be analyzed using a variety of mutation detection techniques for further study.

A schematic overview of the ACE procedure is shown in Figure 1. The initial PCR step utilized a primer set, including a biotin-labeled primer, to amplify the target sequence, which already contained a wild-type-specific restriction enzyme cleavage site. For sequences lacking an available restriction site, dCAPS PCR was performed with a primer set containing a biotin-labeled mismatched primer to create a suitable restriction enzyme cleavage site. Following PCR, the biotin-labeled products underwent wild-type-specific restriction digestion, followed by magnetic isolation using streptavidin-labeled Dynabeads. Through this process, DNA fragments containing mutant sequences were selectively enriched on magnetic beads for further analysis.

### 3.2. Optimization of Streptavidin-Labeled Dynabeads

Four different types of streptavidin-labeled Dynabeads (M-280, M-270, C1, and T1) were evaluated based on their diameters, sedimentation rates, magnetic mobilities, and hydrophilic-hydrophobic properties. To identify the most suitable beads for our application, we tested their binding specificity and interference with downstream mutation detection techniques. Biotin-labeled DNA fragments containing the IDH1-R132H mutation were mixed with unlabeled wild-type IDH1 fragments and purified using each version of Dynabeads. The isolated products were subjected to Sanger sequencing after amplification, showing similar isolation specificity across all bead types (Figure S1A). Additionally, real-time PCR was performed using the magnetically isolated products as templates, revealing that only the M-270 Dynabeads exhibited minimal interference with the PCR reaction (Figure S1B). Based on these results, the M-270 Dynabeads were selected for subsequent experiments.

### 3.3. ACE Specifically Enriched Mutant Sequences of FLT3-TKD with Plasmid Standards

**Figure 2:**
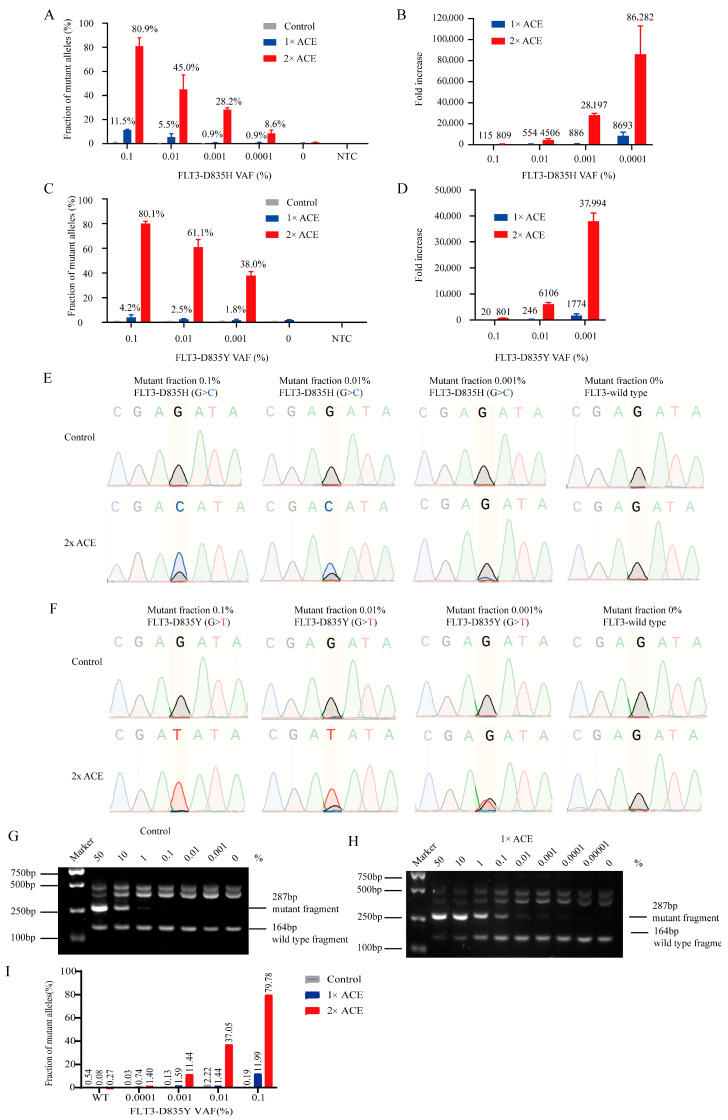
The enrichment efficiency of ACE for FLT3-TKD using plasmid standards. The enrichment efficiency of ACE with plasmid standards for FLT3-D835Y/H was evaluated via quantitative real-time PCR (**A**–**D**), Sanger sequencing (**E**,**F**), AS-PCR (**G**,**H**) and ddPCR (**I**). (**A**,**C**) The percentage of FLT3-TKD tested via quantitative real-time PCR before and after ACE. (**B**,**D**) The fold increase in detection sensitivity following ACE versus control was calculated based on real-time PCR data. (**E**,**F**) The enrichment efficiency of ACE for FLT3-D835Y/H plasmid standards was analyzed via Sanger sequencing. (**G**,**H**) The enrichment efficiency of ACE with plasmid standards for FLT3-D835Y was further analyzed via allele-specific PCR (AS-PCR) and visualized on 3% agarose. (**I**) The enrichment efficiency of ACE for FLT3-D835Y plasmid standards was analyzed via ddPCR.

To evaluate the limit of detection (LOD) of ACE for FLT3-TKD mutations, we created standards by mixing purified plasmids containing FLT3-D835Y/H mutations with wild-type sequences. ACE enrichment significantly enhanced the detection of mutant alleles, as revealed by allele-specific real-time PCR (Figure 2A,C). The sensitivity of real-time PCR increased by approximately 800 to 80,000-fold after two rounds of ACE enrichment (Figure 2B,D). Sanger sequencing confirmed a clear mutation signal in ACE-enriched products, while these signals were negligible in unenriched samples (Figure 2E,F). We also observed a clear linear relationship between the mean cycle threshold (Ct) values and mutant allele fractions using our allele-specific real-time PCR (Figure S2A,B).

Further analysis using nested allele-specific PCR (AS-PCR) demonstrated that without ACE enrichment, AS-PCR had a sensitivity limit of 1% (Figure 2G). However, after one round of ACE enrichment, the sensitivity improved dramatically, reaching 0.0001% (Figure 2H). ddPCR assays confirmed that ACE could be integrated into ddPCR, enhancing the sensitivity of ddPCR for the detection of FLT-D835Y to 0.0001% after two cycles of ACE (Figure 2I), and the representative images of ddPCR were showed in Figure S2C.

### 3.4. ACE Specifically Enriched Mutant Sequences of FLT3-TKD in Blood DNAs

**Figure 3:**
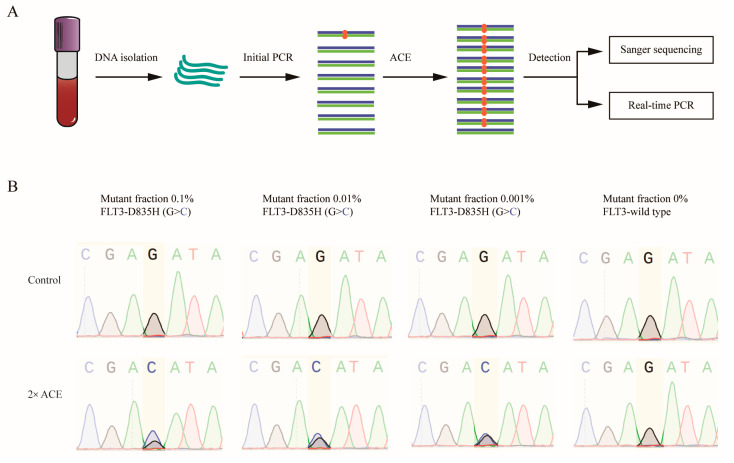
Validation of ACE enrichment efficiency in blood samples from AML patients. (**A**) A flowchart illustrating the procedure of ACE coupled with Sanger sequencing and real-time PCR for mutation detection. (**B**) ACE and Sanger sequencing were used to detect FLT3-D835H mutations in blood DNA samples containing 0%, 0.001%, 0.01%, and 0.1% FLT3-D835H, created by mixing blood DNA from AML patients with FLT3-D835H and healthy donor DNA.

We further tested the LOD of ACE using a blood DNA sample from an AML patient positive for the FLT3-D835H mutation, as confirmed by means of Sanger sequencing (Figure 3A). The blood DNA sample contained a 20% FLT3-D835H mutation. To assess detection limits, we prepared DNA mixtures by combining FLT3-D835H-positive and wild-type DNA at varying ratios, and 1 μg of each mixture was subjected to initial PCR, followed by Sanger sequencing with or without ACE enrichment. Without ACE, Sanger sequencing could detect mutations down to a limit of around 10%, but it failed to detect mutations at 0.1% (Figure 3B, upper panel). However, after ACE enrichment, the detection limit was increased to 0.001% (Figure 3B, lower panel).

### 3.5. ACE Detected MRD in an AML Patient During Complete Remission

**Figure 4:**
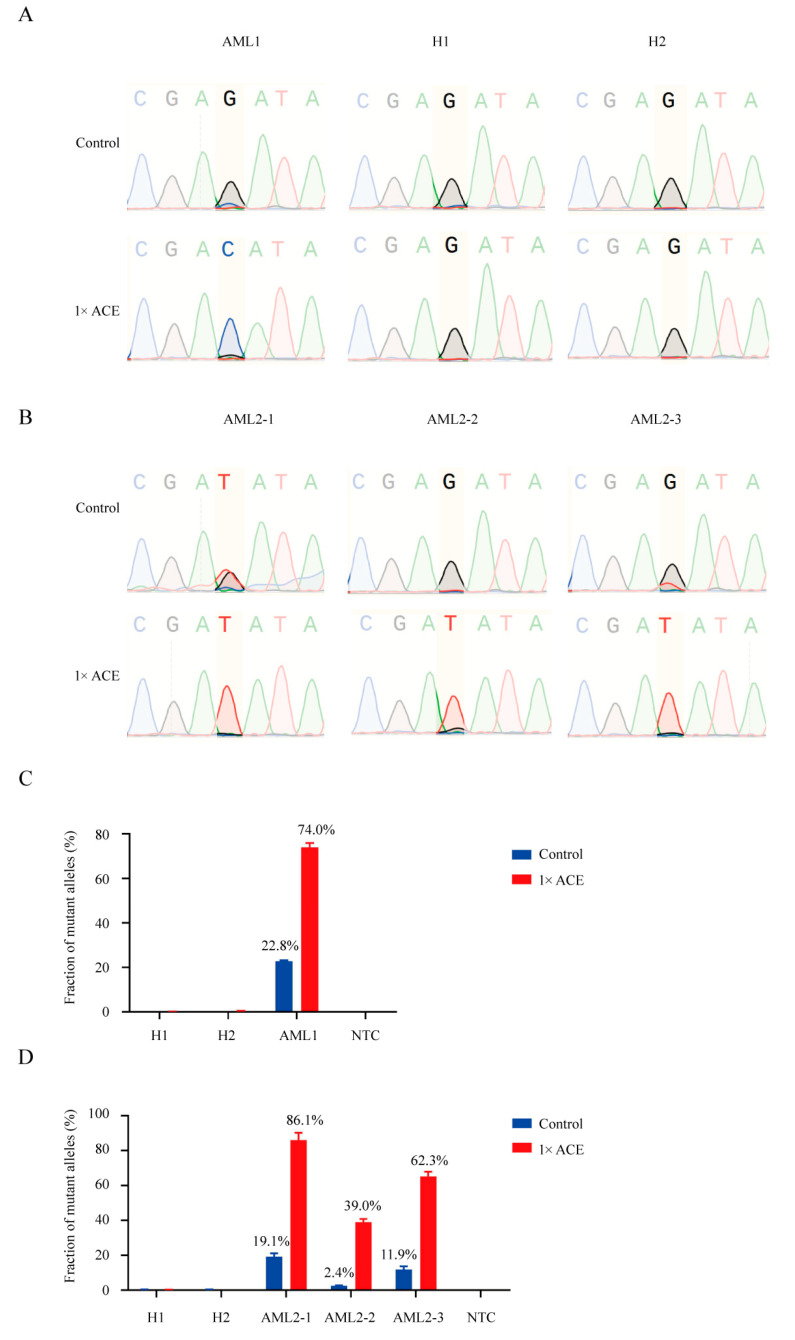
Detection of FLT3-TKD in blood samples from AML patients using ACE coupled with Sanger sequencing and real-time PCR. (**A**) Sanger sequencing of products from an AML patient with approximately 20% FLT3-D835H and two healthy donors, with or without ACE enrichment. (**B**) Sanger sequencing of products from an AML patient with FLT3-D835Y during treatment, with or without ACE enrichment. (**C**) Real-time PCR analysis of products from an AML patient with approximately 20% FLT3-D835H, with or without ACE enrichment. (**D**) Real-time PCR analysis of products from an AML patient with FLT3-D835Y during treatment, with or without ACE enrichment.

We applied ACE in combination with various mutation detection techniques to assess FLT3-TKD mutations and minimal residual disease (MRD) in AML patients. A significantly enhanced FLT3-D835H signal was observed in Sanger sequencing after ACE enrichment, whereas the signal was ambiguous without enrichment (Figure 4A). Additionally, we analyzed blood DNA samples from AML patients, using both Sanger sequencing and real-time PCR before and after ACE enrichment, at different stages of the disease. Notably, in one AML patient, the blood DNA sample was FLT3-D835Y positive at the initial diagnosis (Figure 4B, AML2-1). This patient underwent treatment with decitabine, homoharringtonine, cytarabine, and G-CSF, resulting in complete remission. However, even during remission, a clear mutation signal was detected via Sanger sequencing following ACE enrichment, which was undetectable in standard Sanger sequencing (Figure 4B, AML2-2). Two weeks later, the patient relapsed, as confirmed via Sanger sequencing (Figure 4B, AML2-3). These results were further validated via real-time PCR (Figure 4C,D), indicating that ACE significantly improved the sensitivity of MRD detection in AML. To further validate the clinical application of ACE, we subjected ACE to Sanger sequencing in an AML cohort with 23 patients and found that two AML patients had FLT3-D835Y mutation (Figure S3A). Notably, the FLT3-D835Y signal of AML9 was ambiguous and the FLT3-D835Y signal of AML21 was not observed before ACE enrichment, whereas the signals of FLT3-D835Y mutation were close to 100% after ACE enrichment in both two samples (Figure S3B).

### 3.6. dCAPS PCR for Creation of Wild-Type-Specific Restriction Cleaving Sites

**Figure 5:**
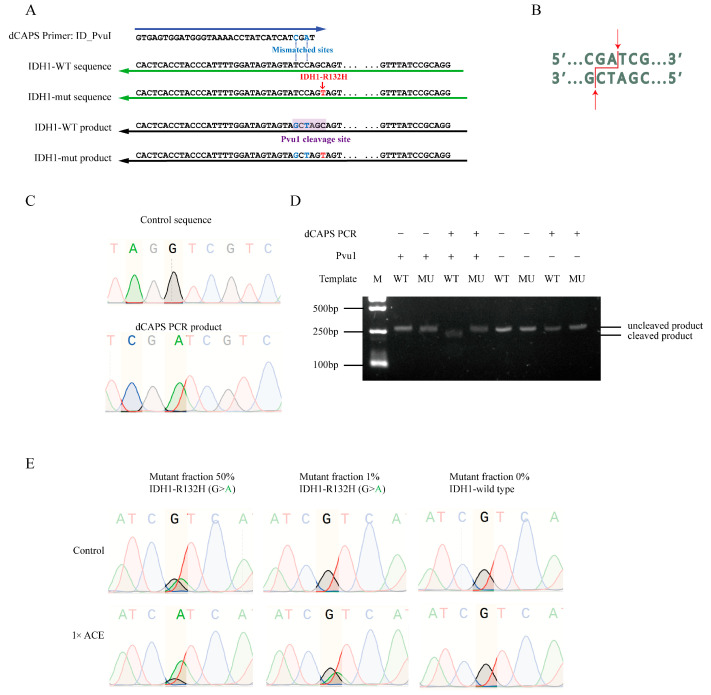
ACE enrichment of mutant alleles of IDH1-R132H without restriction enzyme cleavage sites. (**A**) Schematic representation of dCAPS PCR used to introduce a PvuI cleavage site in wild-type IDH1 sequences, but not in IDH1-R132H mutant sequences. (**B**) Illustration of the PvuI cleavage site in the wild-type sequence. (**C**) Sequence comparison of products generated via regular PCR versus dCAPS PCR. (**D**) Gel electrophoresis analysis of PvuI cleavage efficiency in products generated via regular PCR or dCAPS PCR with wild-type or mutant IDH1 sequences. (**E**) Sanger sequencing of products with or without ACE enrichment in plasmid DNAs containing 50%, 1%, and 0% IDH1-R132H.

ACE-based detection of FLT3-TKD mutations relies on the presence of wild-type-specific restriction enzyme cleaving sites. However, many oncogenic mutations lack such sites. To address this, we tested the feasibility of creating wild-type-specific restriction cleaving sites via dCAPS PCR using mismatched primers. We selected the IDH1-R132H mutation as a model target and successfully introduced BceAI or PvuI cleaving sites into the wild-type sequence using dCAPS PCR with mismatched primers (Figure 5A,B and Figure S4A,B). Sanger sequencing confirmed that these cleavage sites were introduced correctly (Figure 5C and Figure S4C). The dCAPS PCR products derived from IDH-WT sequences were cleavable by PvuI or BceAI as expected, while products from the IDH-R132H mutation remained uncleavable (Figure 5D and Figure S4D). Further, ACE specifically enriched IDH1-R132H mutant alleles from mismatched primer-amplified fragments (Figure 5E and Figure S4E). Notably, PvuI can discriminate and cleave wild-type sequences for both IDH1-R132H and IDH1-R132C, whereas BceAI is specific to IDH1-R132H.

## 4. Discussion

In this study, we developed a novel and ultrasensitive method ACE (Amplifying–Cleaving–Enriching), which depends on wild-type-specific restriction enzyme cleavage and streptavidin–biotin isolation to efficiently enrich rare mutations in AML. Depending on the differences between wild-type and mutant sequences, ACE can utilize a wild-type-specific restriction enzyme to selectively cleave wild-type sequences. The cleaved fragments containing wild-type site are then removed through streptavidin–biotin isolation, thereby preventing wild-type sequences from amplifying in the subsequent PCR amplification. To generate a suitable restriction enzyme cleavage site in sequences lacking an endogenous restriction site, dCAPS PCR was performed using a primer set containing a biotin-labeled mismatched primer. Notably, when designing the dCAPS PCR primer set, the wild-type restriction enzyme cleavage site should be positioned between the biotin modification and the nucleotide polymorphism site, ensuring that the cleaved fragment containing the wild-type site is washed away.

To invalidate the limit of detection of ACE, we generated plasmid standards and DNA mixtures from AML patients for ACE combined with Sanger sequencing, real-time PCR, AS-PCR and ddPCR. ACE significantly enhanced the sensitivity of mutation detection techniques, with around 20–8000 fold improvement after one round of enrichment. Remarkably, two rounds of ACE increased the detection sensitivity of FLT3-TKD mutant sequences by more than 80,000 times in plasmid models. Additionally, we demonstrated the flexibility of ACE by successfully enriching IDH1-R132H mutant sequences, which lack wild-type-specific restriction enzyme cleavage sites. Further, we extended ACE to other driver mutations, including BRAF, KRAS, NRAS, EGFR, JAK2, KIT, and PTPN11, as detailed in Table 1.

Mutations in FLT3 and IDH1/2 are clinically significant therapeutic targets and prognostic biomarkers in AML [7,11,16,17,18,19]. FLT3-TKD mutations confer resistance to treatments like sorafenib and quizartinib due to the constitutive activation of FLT3 [20]. Previous research indicates that FLT3-TKD mutations, often absent at diagnosis, can emerge during treatment, particularly in patients with FLT3-ITD [1,20]. Early and sensitive detection of FLT3-TKD mutations at low levels is thus critical for improving patient outcomes. In contrast, the prognostic role of IDH1/2 mutations in AML remains unclear [21,22]. Studies have linked IDH1 mutations to poorer outcomes, whereas IDH2 mutations may be associated with a more favorable prognosis [23,24]. Targeted therapies, such as enasidenib and ivosidenib, have shown clinical benefits for IDH1/2-mutated relapsed/refractory (R/R) AML patients, significantly improving overall response and complete remission rates. However, drug resistance and relapse are common, underscoring the need for continuous monitoring of minimal residual disease (MRD) through IDH1/2 mutation detection during treatment [11].

Current MRD detection methods in AML, such as next-generation sequencing (NGS) and multicolor flow cytometry (MFC), each have limitations. NGS offers comprehensive mutation profiling but is labor-intensive with longer turnaround times. MFC is faster and more cost-effective but relies on leukemic cell immunophenotypes, which can change during treatment, potentially leading to inaccuracies. Approximately 25% of AML patients with negative MRD assessments still relapse, highlighting the need for improved sensitivity in current MRD detection techniques.

MRD is an independent post-diagnostic prognosticator and has been adopted as a new outcome definition by the European Leukemia Net [25]. Determining the mutational status of NPM1 (and FLT3) is a necessary step for the genetic-based risk categorization of AML, according to the updated 2022 European Leukemia Net (ELN) guidelines [26]. AML relapse and resistance entail a complex interplay between residual leukemic clones and immune cells, which together facilitate immune evasion and disease recurrence—a dimension not captured by the mere quantification of residual leukemia cells. Rapid diagnostic turnaround, well-coordinated logistics, and creative clinical trial designs that can keep up with advancements in MRD-detection technology and related targeted medicines will be critical to the future success of MRD-directed therapy [27,28]. However, the standardization of MRD detection techniques remains incomplete, which currently precludes their validated application as surrogate endpoints for survival in clinical trials and limits the implementation of MRD-guided therapeutic strategies in routine clinical practice.

Our ACE method has the potential to enhance MRD detection when combined with existing technologies. We screened the FLT3-TKD in AML patients at different stages of disease by ACE, and three AML patients were found to have FLT3-TKD that could not be directly confirmed via Sanger sequence. Notably, we detected MRD in DNA samples from an AML patient at the stage of regression through ACE combined with Sanger sequence, failing to be found by standard Sanger sequence or flow cytometry [15], which suggests ACE enhances MRD detection and makes early intervention possible.

Several emerging methods have also demonstrated the ability to detect mutations at low levels (Table 2) [29,30,31,32,33,34,35,36]. These approaches, which rely on novel nucleic acid-editing enzymes or specialized PCR techniques, can be further improved by integrating ACE. Conventional methods like allele-specific PCR and real-time PCR can detect mutations with a sensitivity as low as 1%. Techniques such as BDA and COLD-PCR, while highly sensitive, require precise temperature control [29,30]. Droplet digital PCR (ddPCR), the most advanced method to date, reliably detects mutations with a fractional abundance as low as 0.1%, with reports suggesting detection of fractions as low as 0.01% [37]. In ddPCR, the bulk reaction is divided into thousands of nanoliter-sized droplets before PCR amplification, and then the absolute quantitative results are calculated using Poisson statistics based on the frequencies of positive droplets, which abrogates the need for standard curves. ddPCR achieves more analytical sensitivity and is more precise and accurate due to its functioning principles. Several studies have reported the wide application of ddPCR for tracing tumor-specific mutations in AML for early detection of relapse, including NPM1, IDH1, IDH2, FLT3-TKD, NRAS, JAK2, SRSF2, SF3B1, KIT and other mutations [38,39,40,41,42]. However, these characteristics limit the wide applications of ddPCR for clinical sample testing or large-scale testing: (1) limits of detection have specificity to individual mutations or hotspots, and limits of detection for major assays are no lower than 0.1% [38,43] and (2) high cost is required for the ddPCR platform and associated consumables compared with integrating ACE (ACE integrates into Sanger sequencing, AS-PCR or real-time PCR). Incorporating ACE into ddPCR workflows could further enhance its sensitivity for detecting low-frequency mutations. Notably, the dynamic range of the ddPCR system is from 1–120,000 copies in 20 μL reaction; therefore, the ACE-enriched production should be diluted into a suitable concentration. There are still several limitations to incorporation of ACE and ddPCR: (1) The experimental processing of ddPCR is not simple, and the incorporation work flow takes more time and more procedures, bringing higher risk for contamination; (2) The streptavidin magnetic beads linked to ACE-enriched productions may potentially affect the formation of microdroplets and inhibit amplification reaction, and removal of streptavidin-labeled Dynabeads may further enhance the sensitivity and accuracy.

ACE has a unique advantage in improving sensitivity, and it costs only USD 10–15 to process each sample, taking just 6 h. The use of ACE in clinical settings could enhance diagnostic sensitivity, with cost-effectiveness and time-saving in patient management.

Even though this study highlights the benefits of ACE, there are still certain areas where it has to be enhanced. Increasing the number of ACE cycles could boost detection sensitivity, but doing so would raise the risk of contamination and false-positive results, which should be avoided by using extremely clean experimental procedures. Theoretically, ACE might be used to identify more mutations and illnesses, but further research is still needed. Our investigation only included a small number of AML samples, and a larger sample size in future studies might better illustrate the false-positivity rate and enhance the generalizability.

In conclusion, we have developed a simple, effective method for enriching mutant sequences with significant clinical applications, particularly in managing AML and potentially other cancers. ACE offers an affordable and efficient solution to improve the sensitivity of mutation detection, especially in the context of MRD monitoring and therapeutic resistance.

## Figures and Tables

**Table 1:** Design of mismatched primers for the creation of wild-type-specific cleaving sites for hot-spot oncogenic mutations.

Genes	Amino Acid Changes	DNA Changes	Original Sequences	Derived Sequences	Enzymes	dCAPS Primer (Red Labels Indicate Modifications from the Original Sequences)
*BRAF*	V600E	chr7: g.140753336A>T	ACAGT	ACTAGT	SpeI-HF	AAAAATAGGTGATTTTGGTCTAGCTACTAG
*KRAS*	G12D	chr12: g.25245350C>T	GCTGGT	ACCGGT	AgeI-HF	CTGAATATAAACTTGTGGTAGTTGGAACCG
*NRAS*	Q61R	chr1: g.114713908T>C	TGGACA	TGTACA	BsrGI-HF	GTTTGTTGGACATACTGGATACAGCTGTAC
*EGFR*	L858R	chr7: g.55191822T>G	TGGCCA	-	MscI	-
*EGFR*	T790M	chr7: g.55181378C>T	GCTCATCAC	GTGCATCAC	DraIII-HF	TCTGCCTCACCTCCACCGTGCAGTGCATCA
*JAK2*	V617F	chr9: g.5073770G>T	GGAGTATGTGTCTGT	-	BsaXI	-
*KIT*	D816V	chr4: g.54733155A>T	GAGAC	-	BsmAI	-
*KIT*	N822K	chr4: g.54733174T>G	TTATGT	TTATAA	SspI-HF	ATGGGTACTCACGTTTCCTTTAACCTTATA (reversed)
*PTPN11*	E76K	chr12: g.112450406G>A	TGGCTG	CAGCTG	PvuII-HF	TATGGAGGGGAGAAATTTGCCACTTCAGCT
*PTPN11*	D61Y	chr12: g.112450361G>T	CACTGGTG	CACTGTGTG	DraIII-HF	TCACCCACATCAAGATTCAGAACACTGTGT

**Table 2:** Major characteristics of reported techniques to enrich and detect rare alleles.

Method	Nuclease	Sensitivity	Specificity	Enrichment Efficiency
COLD-PCR [29]	-	0.01% with NGS	Medium	500-fold
BDA [30]	-	0.01% with qPCR	Medium	10,000-fold
HOLMESv2 [31]	Cas12	0.1% with fluorescence detection	High	-
SHERLOCKv2 [32]	Cas12, Cas13 and Csm6	0.6% with fluorescence detection	High	-
PAND [33]	PfAgo	0.1% with fluorescence detection	High	-
Cut-PCR [34]	Cas9	0.01% with NGS	High	18-fold
NAVIGATER [35]	TtAgo	0.01% with XNA-PCR	High	60-fold (0.5%VAF)
A-Star [36]	PfAgo	0.01% with TaqMan qRT-PCR	High	5500-fold
ACE	Restriction Endonucleases	0.0001% with Real-Time PCR/Sanger sequence/AS-PCR/ddPCR	High	80,000-fold

## Data Availability

All data reported in this study can be shared by the lead contact upon request. Any further information required to reanalyze the data reported in this paper is available from the lead contact upon request.

## Supplementary Materials

The following supporting information can be downloaded at: https://www.mdpi.com/article/10.3390/biomedicines13123026/s1, Table S1: The sequences of primers; Figure S1: Optimization of streptavidin-labeled Dynabeads for isolating biotin-labeled PCR products; Figure S2: The enrichment efficiency of ACE for FLT3-TKD using plasmid standards; Figure S3: The application of ACE for screening FLT3-TKD mutations in AML cohort; Figure S4: ACE enrichment of mutant alleles of IDH1-R132H through the creation of a BceAI cleavage site.
