# Supplementary material for: Ultrasensitive Detection of Rare Mutations via Amplifying–Cleaving–Enriching in Acute Myeloid Leukemia
Source: Biomedicines. 2025 Dec 10;13(12):3026. doi: 10.3390/biomedicines13123026 (PMC12731007; doi:10.3390/biomedicines13123026)
Supplement: Supplementary file 1 [file biomedicines-13-03026-s001.zip › biomedicines-3857875-supplementary.pdf]

SUPPLEMENTARY INFORMATION

Ultrasensitive Detection of Rare Mutations Via Amplifying-Cleaving-Enriching in Acute Myeloid Leukemia

Xiaomei Zhuang [1, 3, 4, †], Lingling Ma [1, †], Liuting Yu [1, †], Yuming Zhao [1], Dengyang Zhang [1], Chunmou Li [1], Chaoxing Liu [1], Yan Xiao [1], Zhiguang Chang [1], Shuping Li [2], Chun Chen [1], Yun Chen [1], Guoying Zhou [1], Zhizhuang Joe Zhao [2, *], and Yao Guo [1, *]

1 Pediatric Hematology Laboratory, Division of Hematology/Oncology, Department of Pediatrics, The Seventh Affiliated Hospital of Sun Yat-Sen University, Shenzhen, Guangdong, China

2 Department of Pathology, University of Oklahoma Health Sciences Center, 940 Stanton L. Young Blvd., BMSB 451, Oklahoma City, OK 73104, USA

3 Digestive Diseases Center; The Seventh Affiliated Hospital of Sun Yat-Sen University, Shenzhen, Guangdong, China

4 Guangdong Provincial Key Laboratory of Digestive Cancer Research, The Seventh Affiliated Hospital of Sun Yat-Sen University, Shenzhen, Guangdong, China

***Correspondence:** Dr. Yao Guo (guoy75@mail.sysu.edu.cn), Pediatric Hematology Laboratory, Division of Hematology/Oncology, Department of Pediatrics, The Seventh Affiliated Hospital, Sun Yat-sen University, Shenzhen, 518107, Guangdong, China; Dr. Zhizhuang Joe Zhao (joe-zhao@ouhsc.edu), Department of Pathology, University of Oklahoma Health Sciences Center, 940 Stanton L. Young Blvd., BMSB 451, Oklahoma City, OK 73104 USA.

† These authors contributed equally to this work.

Running title: Ultrasensitive Detection of Mutations Via ACE

Supplementary Tables

Supplementary Table S1. The sequences of primers

Name	Sequence(5’-3’)
F1_5	5’-Biotin-AGAAGCCGCACAAAGAAC
F1_3	5’-CACCCAGCCAATTCACTC
D1_5	5’-TGATGCCACCAACGACCAAGTCA
D1_3	5’-TGTAGGGTGGTACTCAAGCATTGAA
F1_5n	5’-GCACTCCAGGATAATACAC
F1_3n	5’-AGCCCAAGGACAGATGTGATG
F1_mut	5’-ATATGTGACTTTGGATTGGCTCTAT
F1_wt	5’-CATAGTTGGAATCACTCATGATCTC
F1-5H	5’-ATATGTGACTTTGGATTGGCTCCAC
F1-5Y	5’-ATATGTGACTTTGGATTGGCTCCAT
F1-Ym5	5’-ATTGGCTCGATATATCATGAGTG
F1-m3	5’-CCAAAGTCACATATCTTCAC
F1-Hm5	5’-ATTGGCTCGACATATCATGAGTG
D1_Hm5	5’-ATCATAGGTCATCATGCTTATG
D1_Hm3	5’-GATAGGTTTTACCCATCC
ID_BceAⅠ	5’-Biotin-ATCTGCAAAAATATCCCCCGGCTTGTGAGTGGATGGGTAAAACCTATCATCATAGGCC
ID_PvuⅠ	5’-Biotin-GTGAGTGGATGGGTAAAACCTATCATCATCGAT
ID_r	5’-GGACGCCTATTTGTAAGT
F_TAQ_5	CCAGGAACGTGCTTGTCA
F_TAQ_3	GGAAATAGCAGCCTCACA

Probe_DY	TTGGCTCGATATATCATGAG
Probe_WT	TTGGCTCGAGATATCATGAG

Supplementary Figures

Figure S1

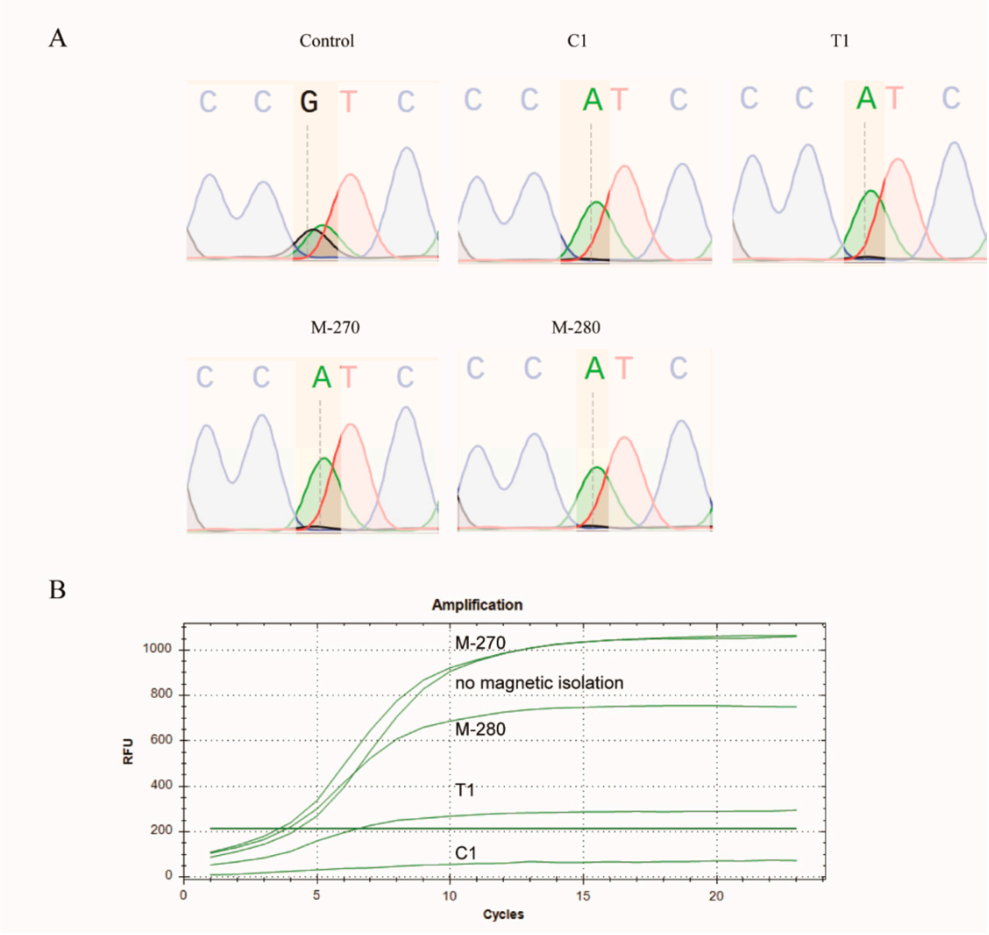

Figure S2

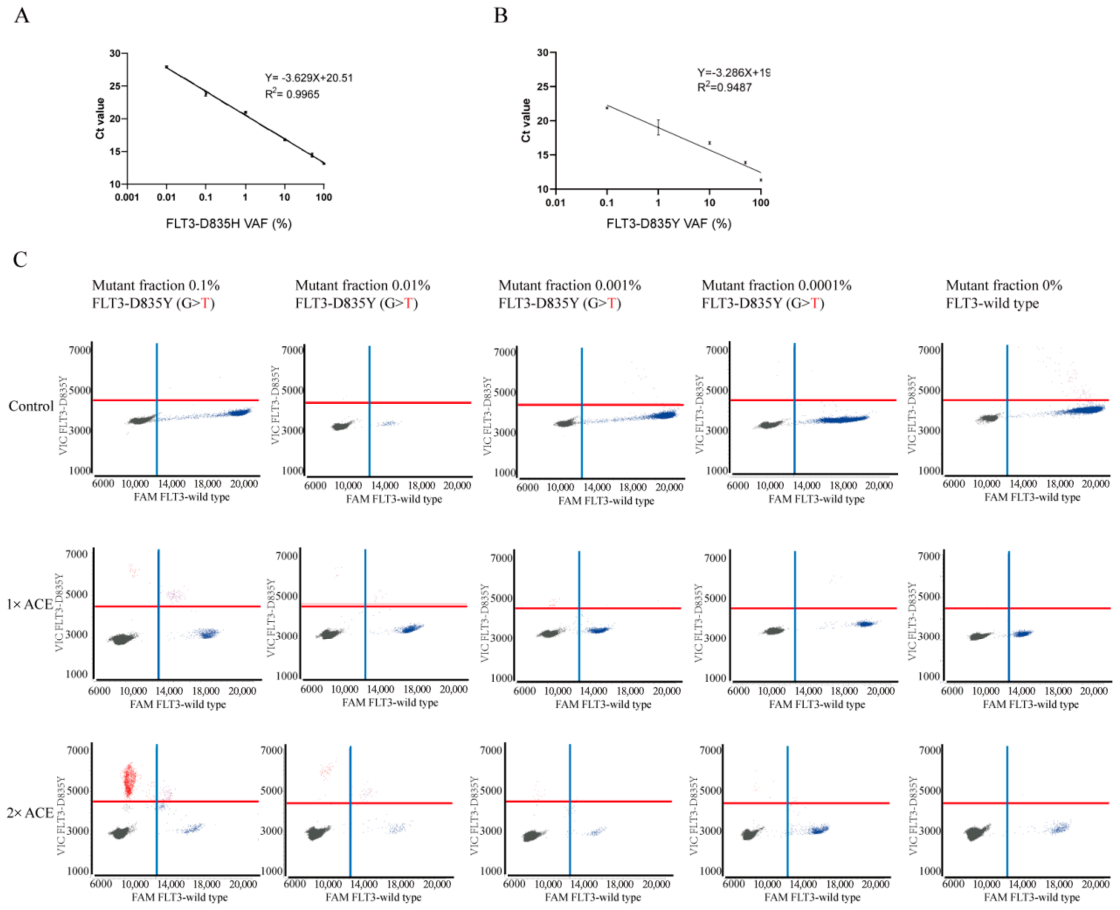
A
Y= -3.629X+20.51
R^2= 0.9965
Ct value
FLT3-D835H VAF (%)
B
Y=-3.286X+19
R^2=0.9487
Ct value
FLT3-D835Y VAF (%)
C
Mutant fraction 0.1%
FLT3-D835Y (G>T)
Mutant fraction 0.01%
FLT3-D835Y (G>T)
Mutant fraction 0.001%
FLT3-D835Y (G>T)
Mutant fraction 0.0001%
FLT3-D835Y (G>T)
Mutant fraction 0%
FLT3-wild type
Control
1× ACE
2× ACE
VIC FLT3-D835Y
FAM FLT3-wild type

Figure S3

A

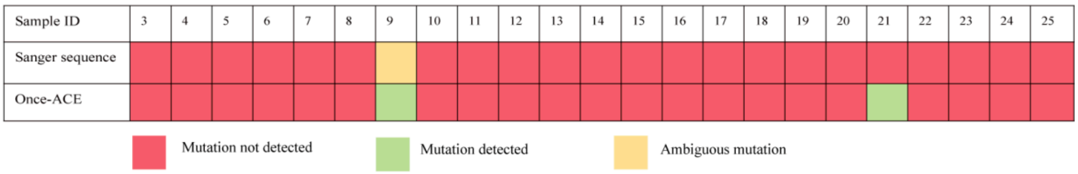
Sample ID
3
4
5
6
7
8
9
10
11
12
13
14
15
16
17
18
19
20
21
22
23
24
25
Sanger sequence
Once-ACE
Mutation not detected
Mutation detected
Ambiguous mutation

B

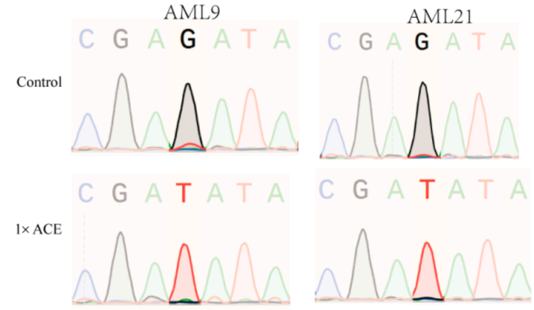
AML9
AML21
Control
1× ACE
C G A G A T A
C G A G A T A
C G A T A T A
C G A T A T A

Figure S4

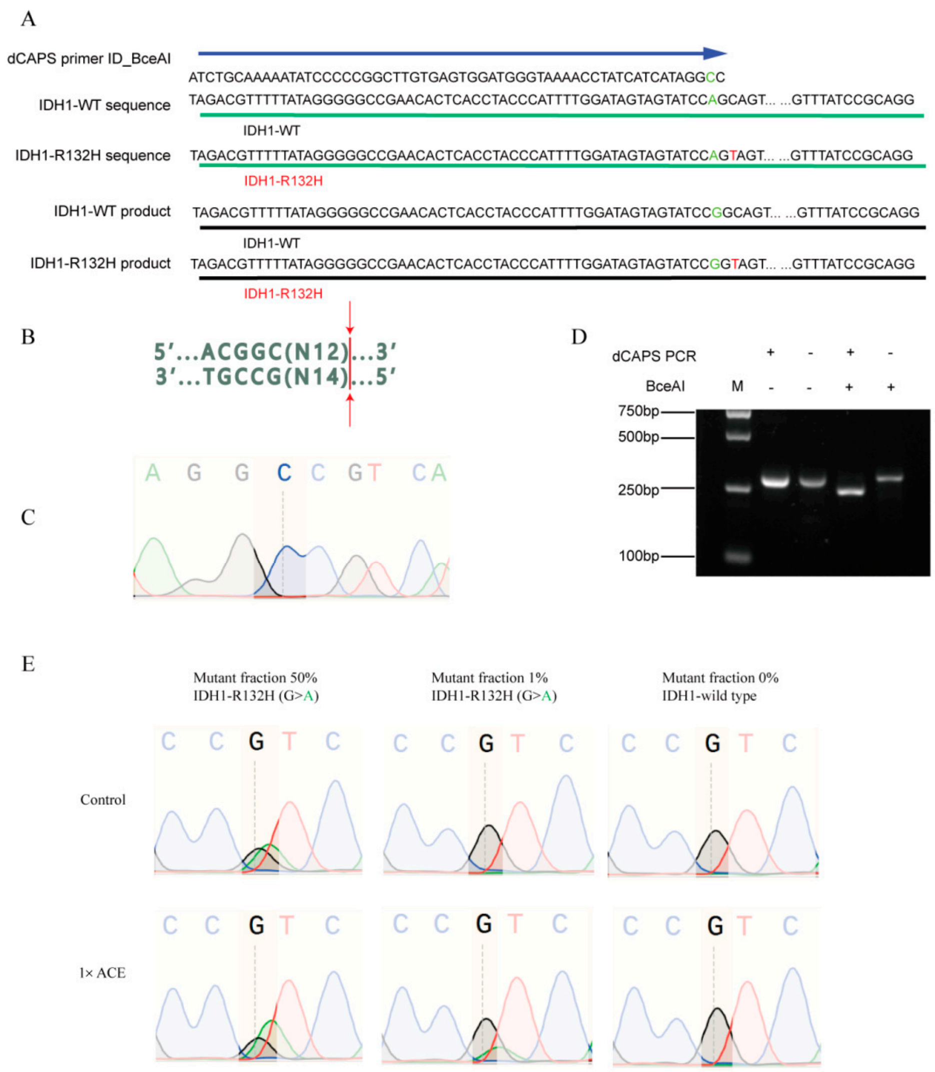
A
dCAPS primer ID_BceAI
ATCTGCAAAAATATCCCCCGGCTTGTGAGTGGATGGGTAAAACCTATCATCATAGGCC
IDH1-WT sequence
TAGACGTTTTTATAGGGGGCCGAACACTCACCTACCCATTTTGGATAGTAGTATCCAGCAGT... ...GTTTATCCGCAGG
IDH1-WT
IDH1-R132H sequence
TAGACGTTTTTATAGGGGGCCGAACACTCACCTACCCATTTTGGATAGTAGTATCCAGTAGT... ...GTTTATCCGCAGG
IDH1-R132H
IDH1-WT product
TAGACGTTTTTATAGGGGGCCGAACACTCACCTACCCATTTTGGATAGTAGTATCCGGCAGT... ...GTTTATCCGCAGG
IDH1-WT
IDH1-R132H product
TAGACGTTTTTATAGGGGGCCGAACACTCACCTACCCATTTTGGATAGTAGTATCCGGTAGT... ...GTTTATCCGCAGG
IDH1-R132H
B
5'...ACGGC(N12)...3'
3'...TGCCG(N14)...5'
C
A G G C C G T C A
D
dCAPS PCR + - + -
BceAI M - - + +
750bp
500bp
250bp
100bp
E
Mutant fraction 50%
IDH1-R132H (G>A)
Mutant fraction 1%
IDH1-R132H (G>A)
Mutant fraction 0%
IDH1-wild type
Control
1× ACE
C C G T C

Supplementary Figure Legends

Supplementary Figure S1. Optimization of streptavidin-labeled Dynabeads for isolating biotin-labeled PCR products. (A) Sanger sequencing results of products isolated using C1, T1, M270, and M-280 Dynabeads from a mixture of 50% biotin-labeled IDH1-R132H sequences and 50% label-free wild-type IDH1 sequences. (B) Assessment of the impact of C1, T1, M270, and M-280 Dynabeads on real-time PCR amplification efficiency.

Supplementary Figure S2. The enrichment efficiency of ACE for FLT3-TKD using plasmid standards. (Ct) values to the rate of FLT3-TKD mutations detected by allele-specific real-time PCR. (A) Correlation between Ct values and the mutation rate of FLT3-D835H. (B) Correlation between Ct values and the mutation rate of FLT3-D835Y. The no-template control consisted of ddH_2O. (C) The representative ddPCR images. Error bars represent the mean ± S.D., n=3.

Supplementary Figure S3. The application of ACE for screening FLT3-TKD mutations in AML cohort. (A) Summary of screening results. Red boxes represent that mutation was not detected; Yellow boxes represent that mutation was ambiguous; And green boxes represent that mutation was detected. (B) Two AML patients were found FLT3-D835Y mutation by ACE, and the sanger sequence results before and after ACE enrichment were presented.

Supplementary Figure S4. ACE enrichment of mutant alleles of IDH1-R132H through the creation of a BceAI cleavage site. (A) Schematic representation of dCAPS PCR to introduce a BceAI cleavage site in sequences containing wild-type IDH1, but not IDH1-R132H. (B) Illustration of the BceAI cleavage site. (C) Sequences of PCR products generated using dCAPS PCR. (D) Gel electrophoresis analysis of BceAI cleavage efficiency in products from regular

PCR or dCAPS PCR with wild-type IDH1 sequences. (E) Sanger sequencing of products with or without ACE in plasmid DNAs containing 50%, 10%, 1%, and 0% IDH1-R132H.
